# Evaluation of community-based continuous distribution of long-lasting insecticide-treated nets in Toamasina II District, Madagascar

**DOI:** 10.1186/s12936-017-1985-7

**Published:** 2017-08-10

**Authors:** Celine Zegers de Beyl, Albert Kilian, Andrea Brown, Mohamad Sy-Ar, Richmond Ato Selby, Felicien Randriamanantenasoa, Jocelyn Ranaivosoa, Sixte Zigirumugabe, Lilia Gerberg, Megan Fotheringham, Matthew Lynch, Hannah Koenker

**Affiliations:** 1 Malaria Consortium, London, UK; 2 Tropical Health, LLP, Montagut, Spain; 3 Johns Hopkins Center for Communication Programs, Baltimore, MD USA; 4 Malaria Consortium, Kampala, Uganda; 5 Catholic Relief Services, Antananarivo, Madagascar; 6 President’s Malaria Initiative, Antananarivo, Madagascar; 7 President’s Malaria Initiative, US Agency for International Development, Washington, DC USA

## Abstract

**Background:**

Continuous distribution of insecticide-treated nets (ITNs) is thought to be an effective mechanism to maintain ITN ownership and access between or in the absence of mass campaigns, but evidence is limited. A community-based ITN distribution pilot was implemented and evaluated in Toamasina II District, Madagascar, to assess this new channel for continuous ITN distribution.

**Methods:**

Beginning 9 months after the December 2012 mass campaign, a community-based distribution pilot ran for an additional 9 months, from September 2013 to June 2014. Households requested ITN coupons from community agents in their village. After verification by the agents, households exchanged the coupon for an ITN at a distribution point. The evaluation was a two-stage cluster survey with a sample size of 1125 households. Counterfactual ITN ownership and access were calculated by excluding ITNs received through the community pilot.

**Results:**

At the end of the pilot, household ownership of any ITN was 96.5%, population access to ITN was 81.5 and 61.5% of households owned at least 1 ITN for every 2 people. Without the ITNs provided through the community channel, household ownership of any ITN was estimated at 74.6%, population access to an ITN at 55.5%, and households that owned at least 1 ITN for 2 people at only 34.7%, 18 months after the 2012 campaign. Ownership of community-distributed ITNs was higher among the poorest wealth quintiles. Over 80% of respondents felt the community scheme was fair and simple to use.

**Conclusions:**

Household ITN ownership and population ITN access exceeded RBM targets after the 9-month community distribution pilot. The pilot successfully provided coupons and ITNs to households requesting them, particularly for the least poor wealth quintiles, and the scheme was well-perceived by communities. Further research is needed to determine whether community-based distribution can sustain ITN ownership and access over the long term, how continuous availability of ITNs affects household net replacement behaviour, and whether community-based distribution is cost-effective when combined with mass campaigns, or if used with other continuous channels instead of mass campaigns.

## Background

The distribution of long-lasting, insecticide-treated nets (ITNs) is a recognized tool for reducing the transmission of malaria in malaria-endemic countries. Initial distributions focused on vulnerable pregnant women and children under 5 years old to reduce mortality, using distribution through antenatal and vaccination clinics [1–5], social marketing [6–8] and targeted mass campaigns [9–18]. Since WHO’s call for universal ITN coverage [19], over 1 billion ITNs have been distributed through mass campaigns [20], generally implemented every 3 years, which replaces all existing ITNs as accounting for existing ITNs in households is not operationally practical [21]. A recent study found that 68% of the 40% reduction in malaria incidence between 2010 and 2015 could be attributed to ITNs [22].

Malaria is endemic in 90% of Madagascar, while the entire country is considered to be at risk for malaria [23]. Prior to 2010, the Madagascar Ministry of Health, supported by international partners and donors, focused ITN distribution strategies on key at-risk populations, specifically pregnant women and children under 5 years old, through antenatal care (ANC) and immunization clinics. Highly subsidized nets were also available through social marketing channels [23]. The 2007 mass campaign, integrated with measles vaccination, mebendazole and vitamin A, distributed more than 1.5 million ITNs to children and pregnant women, resulting in 77% household ownership of at least 1 ITN [9]. The 2009–2010 mass campaign aimed at delivering 2 ITNs per household [24]. Post 2010, the Madagascar National Strategic Plan for Malaria shifted from targeted distribution to mass distribution, with a goal of universal coverage with 1 ITN per 2 persons [23]. In late 2012, through a mass distribution campaign implemented by the National Malaria Control Programme (NMCP) and partners, 5,614,456 nets were distributed in 61 districts in order to achieve universal ITN coverage [25]. However, it is clear that ITN ownership and access decline between campaigns due to net loss, wear and tear, population movements, and births of new family members, reducing ITN coverage to levels as low as 40% before the next campaign [26]. In order to maintain universal coverage more consistently, other mechanisms of ITN distribution are needed [27].

WHO recommends that countries that have strengthened routine ITN distribution through health facilities explore ways to move away from mass campaigns and maintain universal coverage through a combination of health facility distribution and continuous distribution (CD) through schools and/or community-based channels [28]. However, evidence showing that these combinations can maintain high levels of ITN ownership and access in between, or in the absence of, mass campaigns is limited. Therefore, a community-based distribution system was piloted in Tomasina II District on the east coast of Madagascar in 2013–2014. This scheme was conceived as a way to provide families with the opportunity to obtain new nets when they need them, through existing community channels. The goal of the pilot was to maintain household ownership of at least 1 ITN at or above 90%.

## Methods

### Study site

Madagascar has an extensive channel of *agents communautaires* (community agents), who provide health education, prevention services, and limited medical care to members of their communities. These community agents formed the backbone of the community-based ITN distribution pilot.

Toamasina II District, an area of high malaria endemicity, was selected for the pilot based on its inclusion in the 2012 mass campaign, a population size appropriate for the available number of ITNs for the pilot, non-government organization (NGO) presence, and because it includes urban, peri-urban and rural areas as well as a mix of accessible and hard-to-reach communities. This area is made up of 170 *fokontanys* (communities) from 17 communes. The total population in 2012 was estimated to be 235,250 residents.

ITN requirements for the district were calculated using NetCALC [29], an Excel-based open source modelling tool. Based on existing ITN coverage and expected median net lifespan, the model estimated that the quantity of ITNs needed to maintain 90% ownership of at least 1 ITN in households in Toamasina II for the first year of the project (2013–2014) was 43,500. The USAID-funded DELIVER project procured the ITNs.

### Programme description

Implementation of this community based distribution programme began in September 2013. It was based on a ‘push–pull’ system, where ITNs were delivered or ‘pushed’ to a nearby hub. Distribution was then driven or ‘pulled’ by demand from households. In each *fokontany,* 2 community agents received stocks of coupons to distribute to pregnant women receiving antenatal clinic (ANC) consultations, to children receiving measles vaccination, and to the general public who were eligible through established criteria. The criteria agreed upon by the NMCP and stakeholders were:

1. Woman is pregnant
2. Child has completed vaccination (9 months)
3. Sleeping space not covered
4. Newly married couple
5. Just moved to the village
6. Hole in net (2 head-size holes or 5 fist-size holes).

**Fig. 1:**
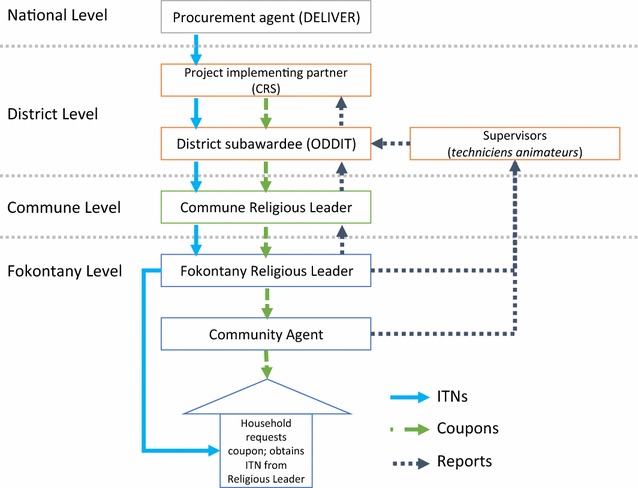
The design and structure of the community-based ITN distribution scheme

If a community member needed an ITN, they would approach a community agent to get a coupon. The community agent would evaluate their need according to the criteria, then give them a coupon. Households then redeemed coupons for ITNs at distribution points managed by local religious leaders, usually the leader’s home. The overall process is illustrated in Fig. 1.

To pro-actively provide ITNs to vulnerable pregnant women and infants, community agents were asked to distribute coupons for ITNs and health record books to pregnant women on their first ANC visit and to mothers or guardians of children completing their vaccinations. Primary healthcare workers in government facilities also told mothers or guardians of vaccinated children to obtain a coupon from their community agent in order to obtain an ITN.

Copies of the coupon were retained by the community agent, the recipient and the religious leader. The community agent and religious leader also recorded coupon distribution in their respective record books, and the woman’s or child’s health card was marked at receipt of coupon and at receipt of an ITN.

The *Organe de Développement du Diocèse de Toamasina* (ODDIT) and Catholic Relief Services trained a total of 524 religious leaders and community agents. Several social and behaviour change communication (SBCC) activities were implemented to promote effective participation in the pilot. Job aids were developed for community agents for quick reference on the distribution and monitoring processes. Two radio spots were developed with Population Services International Madagascar, and aired on local radio stations for 6 months to inform the population on malaria prevention and the logistics of the new distribution system. Community agents and religious leaders used interpersonal communication to discuss eligibility and appropriate net use and care with beneficiaries.

### Pilot monitoring

Eight *techniciens animateurs* (technical advisors) from ODDIT held meetings every 3 weeks with community agents and religious leaders to collect coupons, reports and resolve problems. The number of coupons requested, distributed and redeemed were compiled into monthly reports by ODDIT and Catholic Relief Services (Fig. 1). From this information, minor additional supervision and supplementary trainings on filling out reporting forms were incorporated into the scheme. A mid-term process evaluation was conducted by CCP, CRS, ODDIT, and PSI, which also identified minor challenges (related to timeliness of reporting; delays between training workshops and first delivery of ITNs; and some stock outs of ITNs) and improved coordination.

### Endline survey

In June 2014, after 9 months of pilot implementation, an endline survey was conducted to evaluate the results of the community-based continuous-distribution pilot programme in order to determine if it maintained the same ITN ownership and access rates achieved by the universal coverage campaign. The community distribution system began in September 2013, and the survey began 9 months later in June 2014.

### Study design

A representative sample of Toamasina II was obtained using a cross-sectional survey with two-stage cluster sampling. Clusters were defined as *fokontanys* and 45 were selected using probability proportionate to size. In each cluster, 25 households were then selected by first enumerating all households in the cluster (sub-dividing into sections for large clusters), and then using simple random sampling from the list of enumerated households. If no one was present in the household, at least 3 visits were attempted in order to reach someone to interview before discarding the household from the sample without replacing it. The sample size was calculated based on an alpha error of 95%, beta error of 80%, a design effect of 2.0, non-response rate of 7%, and an expected increase in household ownership of at least 1 ITN from 53 to 60% by the end of the pilot. A structured questionnaire, based on the Malaria Indicator Survey with additional questions on processes specific to the community distribution and social and behaviour change communication (SBCC) activities, was used to gather data. The primary respondent was the head of the household or his/her representative and the person who collected the ITN at the distribution point.

### Data management and analysis

Double-entry of all records was done using EpiData software and the two databases were subsequently compared. Any potential conflicting observations were verified using the original paper questionnaire. Data were then transferred to the statistics software Stata^®^ 12.0 for further consistency checks and preparation for analysis. The analysis approach was ‘intent-to-treat’, meaning that every sampled household was included regardless of the distribution of ITNs in the selected households. Primary outcome measures were those of net and ITN ownership as recommended by the Roll-Back Malaria Monitoring and Evaluation Reference Group (RBM-MERG) namely the ‘proportion of households with any ITN’, the ‘proportion of households with at least 1 ITN for every 2 people in the household’ (considered to be enough so that every member could use an ITN) and the ‘proportion of the population with access to an ITN within the household’ assuming each ITN is used on average by 2 people [30]. Chi squared test was used for dichotomous or grouped variable comparisons and Student’s *t* test or non-parametric Kruskal–Wallis test for continuous variables always adjusting for the cluster-design of the survey, using the “svy” command family in Stata. Counterfactual ownership coverage was calculated by using only nets identified as from the campaign or the continuous distribution, respectively.

### Ethical considerations

Individual verbal informed consent was obtained from all respondents before interviews were conducted. Ethical clearance for the study was provided by the Madagascar Ministry of Health as well as the Institutional Review Board of the Johns Hopkins Bloomberg School of Public Health (IRB No: 5683).

## Results

### Coupons and ITNs issued

**Fig. 2:**
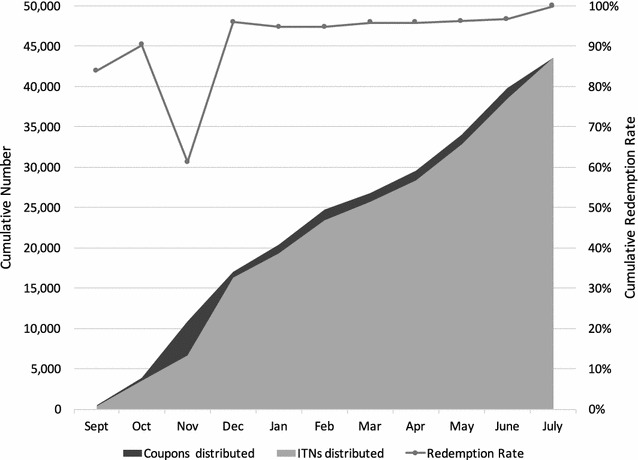
Cumulative coupons and ITNs issued and cumulative redemption rate

Over the 9-month pilot a total of 43,500 coupons were issued and 43,498 ITNs were issued, for a total redemption rate of 99.9%. As shown in Fig. 2, the redemption was steady except for a drop in November, reflecting the delayed delivery of the second shipment of ITNs. The reason for giving the coupon was recorded; overall, 36.4% were issued for a non-covered sleeping space, 27.9% for a torn or stolen net, 18.2% were issued for a vaccinated child, 10.2% for a pregnant woman, and 7.1% for newly married couples or those arriving in the *fokontany*.

### Evaluation survey

Of the 1125 households targeted for the survey, 1121 were interviewed. The majority (81.6%) of households surveyed had a male head of the household with an average age of 42.4 years. There was an average of 4.2 (ranging from 1 to 15) people per household. Overall, 43.6% of households had at least 1 child under 5 years old, and 5.9% included a pregnant woman. Households had on average 1.4 rooms used for sleeping and 2.1 beds, and the estimated ratio of people per bed was 2.1 (ranging from 1 to 8). The distribution of the sampled population by age and by gender was similar to the age pyramid of sub-Saharan African countries, with children under 5 representing 13.2% and children under 15 representing 42.8% of the sampled population. Access to safe water was limited (20.8%), but over 80% of households had access to latrines. Overall, 98.5% of people in households on the day of the survey were usual residents and 97.6% had stayed in the house the previous night. Household characteristics are provided in Table 1.

**Table 1:** Household characteristics

Category/variable	Endline estimate	95% CI
Demographics		
Average household size (persons)	4.2	4.0–4.4
Household headed by female	18.4	15.6–21.5
Mean age of head of household (years)	42.4	41.2–43.6
Population <5 years	13.2	11.9–14.7
House characteristics		
Thatch or grass roof	80.7	75.7–85.0
Wood/bamboo walls	50.5	43.0–58.0
Firewood primary fuel for cooking	87.0	79.6–92.0
Average persons/sleeping place	2.1	2.0–2.2
Education of head of household		
Non-literate	17.7	14.5–21.5
Some primary	59.7	55.8–63.4
Some secondary	20.9	17.1–25.3
Secondary or higher	22.6	18.4–27.5
Water and sanitation		
Household with access to safe water	20.8	11.9–33.9
Household with access to any latrine	86.8	79.0–92.0
Household assets		
Household owns any radio	54.8	50.8–58.8
Household owns any mobile phone	19.0	14.2–24.9
Household has any means of transport	27.4	21.1–34.9

### ITN ownership

**Fig. 3:**
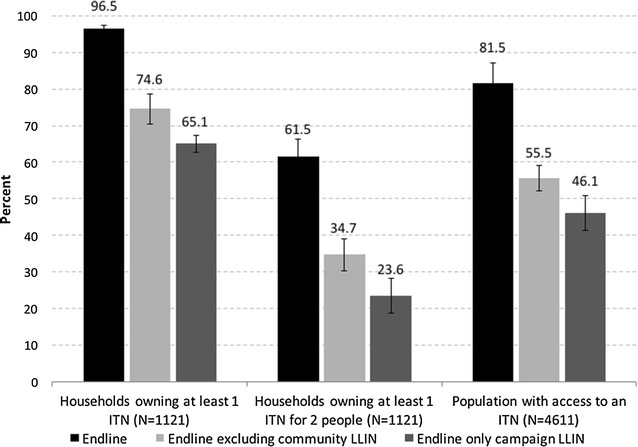
ITN indicators at endline (*black*) and calculated excluding ITNs received through the community system (*light grey*) and considering only ITN from the campaign (*dark grey*)

Out of the 2656 nets present in the sampled households, 89.3% were LLIN, based on the brand, and one was claimed to have been dipped in the last 12 months. As shown in Fig. 3, on the day of the survey, 96.5% of households owned at least 1 ITN, 81.5% of the population had access to an ITN, and 61.5% owned at least 1 ITN for every 2 household members. Without the ITNs provided through the community channel, an estimated 74.6% of households would have owned at least 1 ITN, 55.5% of the population would have had access to an ITN, and 34.7% of households would have owned 1 ITN for 2 people. Furthermore, if only the LLIN remaining from the campaign were considered, the coverage a year-and-a-half after the 2012 campaign was 65.1, 46.1 and 23.6%, respectively.

**Fig. 4:**
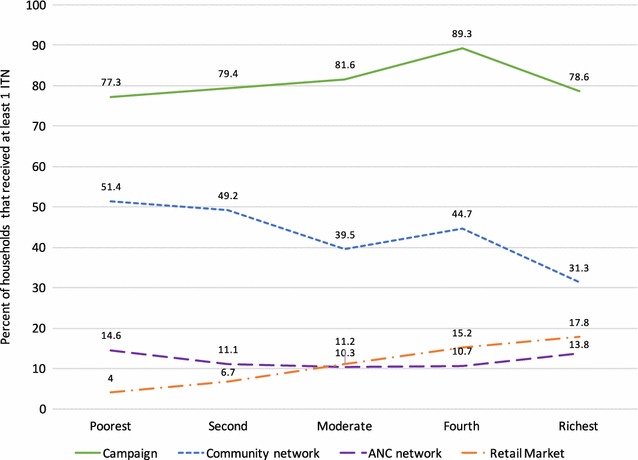
Proportion of households that received at least 1 ITN from different channels

The reach of each ITN distribution channel was defined by the percentage of households that received at least 1 net from the different channels. Overall, 81.2% of households reported to have received at least 1 ITN from the December 2012 campaign. Among the other 4 channels, 43.2% had at least 1 ITN from the community distribution, 12.1% had 1 from the ANC, 11.0% had at least 1 from the retail market (shop/pharmacy/market), and 11.2% from other sources (family/friends, NGO, etc.). Wealth quintile was not associated with campaign or ANC ITN ownership (p > 0.05), shown in Fig. 4. In contrast, poorer wealth quintiles had higher community ITN ownership (p = 0.004), while ownership of ITNs from the private sector increased with increasing wealth (p = 0.0001).

### System effectiveness of the community-based distribution

Out of the 1121 households sampled, 71.4% had been informed about the community distribution of ITNs; among these, 65.5% said that they passed the information on to other families. Overall, 54.1% (606) of sampled households contacted a community agent or religious leader to obtain a new ITN. Out of these 606 households, 84.5% (512) received a coupon from the community agent. The majority (81.9%) of the 512 households that received a coupon went to exchange it and received at least 1 ITN; 11.1% received an ITN without going to the community leader in person to exchange the coupon, suggesting that they gave their coupon to another person to exchange for a net. A few households (1.4%) went to see the community leader but did not receive an ITN, while 5.6% received a coupon from the community agent but did not go to see the leaders and therefore did not receive an ITN. Eight households (0.7%) reported receiving an ITN without having received a coupon. Consequently, the community distribution covered 43.2% of all sampled households, or 484 families. Overall, the distribution process was not associated with socio-economic status once the community agent was contacted. However, requesting a new net was highest in the two lowest wealth quintiles and lowest in among the richest households resulting in an overall pro-poor effect (p = 0.002). Failure to redeem a coupon was also higher in the wealthier quintiles. Table 2 details the effectiveness of the community-based distribution process.

**Table 2:** System effectiveness of the community-based distribution process

Socio-economic quintile	Contacted a community agent or religious leader to acquire an ITN N = 1121 (%)	Received a coupon N = 606 (%)	Among the households that received a coupon N = 512				Did not receive a coupon and received 1 ITN N = 1121 (%)	Received at least 1 ITN N = 1121 (%)
			Went to exchange it/received 1 ITN (%)	Did not go to exchange it/received 1 ITN (%)	Went to exchange it/did not receive an ITN (%)	Did not go to exchange it/did not receive an ITN (%)		
Poorest	59.3	90.3	81	14.9	–	4.1	–	51.3
Second	63.9	81.9	82.9	10.3	2.5	4.2	0.4	49.2
Moderate	50.7	79.6	85.5	7.8	–	6.7	1.8	39.5
Fourth	54.1	85.9	85.6	8.6	1.9	3.8	0.9	44.6
Richest	42.4	84.2	72.5	13.7	2.5	11.2	0.4	31.3
Total % (n)	54.1 (606)	84.5 (512)	81.9 (419)	11.1 (57)	1.4 (7)	5.6 (29)	0.7 (8)	43.2 (484)

**Fig. 5:**
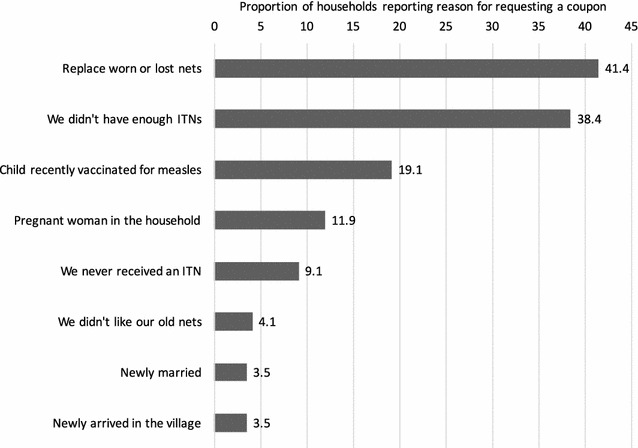
Reasons for requesting coupons from the community agent (n = 606; multiple responses possible)

Survey respondents’ reasons for requesting coupons were recorded, shown in Fig. 5. These followed the same pattern as reasons recorded through the monitoring system. The primary reasons were to replace worn or lost nets or because the household did not have enough ITNs (i.e. they had uncovered sleeping spaces); 67.2% of households reported one of these two reasons. Nearly 20% of households reported having a child recently vaccinated for measles, or a pregnant woman in the household (11.9%). Just under 10% of households reported never having received ITNs. Less than 5% of households reported requesting coupons because they met the eligibility criteria of being newly married or newly arrived in the community.

### Use of ITNs the night before the survey

Overall, 71.2% of the population had used an ITN the night before the survey. Among households with enough ITNs for every member, 85.8% had slept under an ITN. However, use increased with age (p < 0.0001) being 48.1% among children under 5, 67.2% at age 5–14 years and 79.0% for persons 15 years and older. For households with enough ITN for all members the age difference was less pronounced with 72.5, 83.3 and 89.3%, respectively, but was still statistically significant (p < 0.001). There was no difference in use between males and females if there were enough ITNs. However, if there not enough, males were somewhat more likely to use an ITN (59.0 vs 52.4%, p = 0.008). There was no observed increased use for currently pregnant women (n = 68). The ratio of population using an ITN to those with access to an ITN was 0.85.

Out of all the ITNs found in the households, 70.0% were hung up; 5.1% were still in their original package. Respondents reported that 72.6% of ITNs had been used every night the week before the survey, and that 90% of nets had been used the night before. Among the reasons for a net not being used, 47.0% were because the net was worn out or too dirty, 11.2% because the net was not needed, 8.2% because the usual user was not there or the net was not available, and 7.8% because there was not enough space to hang the net.

When asked if a net was slept under by any person in the household the night before, it was found that ITNs were more likely to be used compared to untreated nets (91.6 vs 75.3%, p < 0.05).

### Social and behaviour change communication (SBCC)

SBCC activities contributed significantly to household awareness of the pilot. Overall, 71.4% of respondents reported hearing about the community distribution programme. The three main sources of information were the community agent (44.2%), the radio (37.9%) and healthcare professionals (35.7%). Family and friends were also a significant source of information (18.7%).

**Fig. 6:**
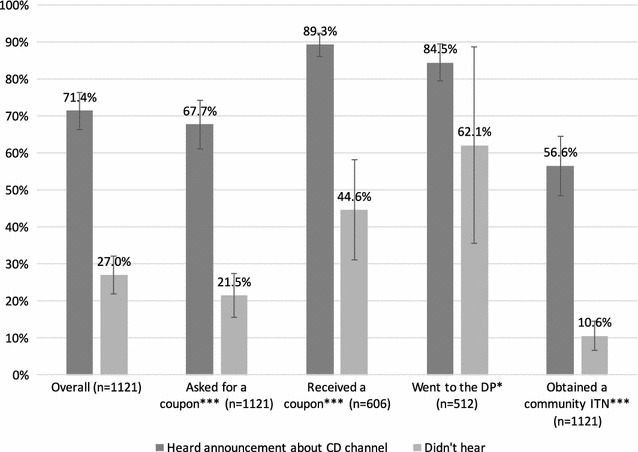
Households that heard about the continuous distribution channel in relation to participation in the continuous distribution channel

SBCC activities had a significant impact on the behaviour of sampled households. Households that had heard about the CD programme were significantly more likely to have asked for a coupon than those who did not receive information (Fig. 6).

**Fig. 7:**
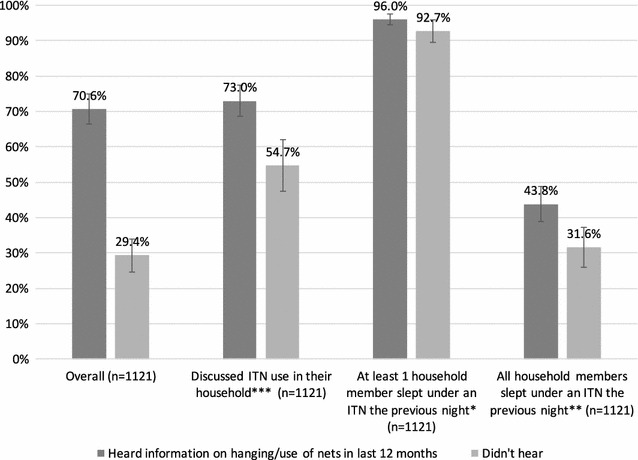
Households that received any information on hanging or use of mosquito nets in the past 12 months in relation to discussion of and use of ITNs

Separately, respondents were asked if they had heard information on hanging or use of mosquito nets in the last 12 months. Exposure to these messages was associated with increased discussion of net use in the household, and having at least one or all family members sleeping under an ITN the night before (Fig. 7).

### Perceptions of the community-distribution scheme

**Fig. 8:**
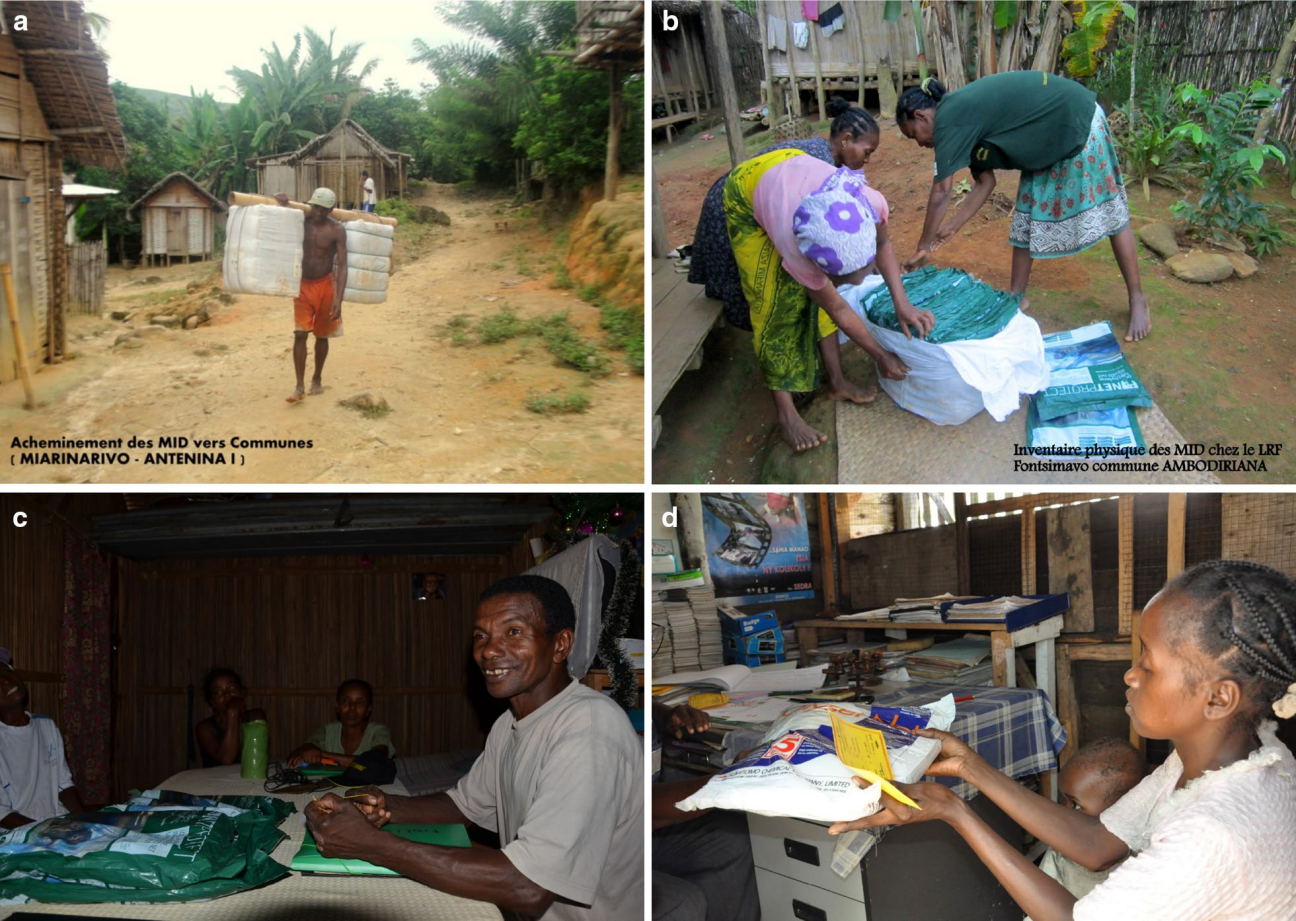
**a** Carrying bales of ITNs from district to commune level in Miarinarivo, Antenina 1. **b** Physical inventory of ITNs at the religious leader’s house in Fontsimavo, Ambodiriana. **c** Religious leader with ITNs during supervision visit. **d** Exchange of coupon for ITN

Generally, the majority of households were satisfied with the service offered (summarized as ‘positive’, ‘acceptable’ and ‘negative’ attitudes in Table 3); 78.1% felt that the distance to travel for the community distribution was “very short”, 84.3% found the coupon exchange service to be “very simple” and 83.0% found that the community agent was “very accessible”. Respondents largely felt that the programme’s criteria for obtaining a new ITN were “very fair” (43.5%) or “reasonably fair” (43.1%). Only 6.1% (37 households) felt the criteria were unfair. Figure 8 illustrates four elements of the CD scheme.

**Table 3:** Coupon-requesting household attitudes towards community distribution (N = 606)

	“What is your opinion about?”			
	The distance to travel (%)	The coupon exchange service (%)	Criteria required to obtain an ITN (%)	Accessibility of the community agent (%)
Positive	78.1	84.3	43.1	83
Acceptable	11.9	7.4	43.5	9.6
Negative	9.5	1	6.1	0.8
No opinion	–	2.3	–	–
Do not know	0.5	5	7.3	6.6
Positive	78.1	84.3	43.1	83

## Discussion

Eighteen months after the last mass campaign, and following the 9-month community-based ITN distribution pilot programme, population ITN access was over the target level of 80%. Nearly 100% of households owned at least 1 ITN, and 61% owned at least 1 ITN for 2 people. While a baseline comparison was not possible, by excluding ITNs obtained from the community distribution from the analysis, it can be estimated that had the pilot not occurred, only 55.4% of the population would have had access to an ITN, and only 18.8% of households would have owned 1 ITN for every 2 people, far below universal coverage targets. Two-thirds of households reported requesting a coupon because they did not have enough ITNs or because their ITNs had worn out or been lost; this suggests coverage had indeed declined since the campaign.

These results demonstrate that a combination of mass distribution campaigns and ongoing continuous distribution can achieve current targets for population ITN access. Achieving targets on universal access remains illusory for most countries. The results of this pilot demonstrate it is achievable with a combination of different distribution approaches. It is likely that no single distribution channel will achieve coverage and access targets in the large majority of settings.

However, if continuous systems can successfully sustain high levels of ITN access up until the next scheduled campaign, the campaign could be potentially replacing a higher proportion of relatively new ITNs. This may be considered oversupply or over-allocation. As Bhatt et al. showed, due to repeated mass campaigns, 22–32% of ITNs were estimated to be over-allocated in 2013 [31], and this rate is likely to increase if robust continuous channels are added to repeated mass campaigns. In contrast, data on robust, longer-term continuous distribution approaches are very limited; it is as yet unknown whether these approaches can achieve and sustain coverage and access over the longer term. Furthermore, the cost of determining who really requires a replacement net and who does not may ultimately cost more than the costs involved in over-supplying some ITNs to a community. Finding the best combinations of channels to distribute enough ITNs to reach coverage targets, but not too many, is one of many challenges faced by the global malaria community.

The percentage of households that contacted a community agent was higher among the two poorest quintiles and lowest among the richest quintile, suggesting community-based distribution can succeed in reaching those in greatest need. This is in contrast to findings from South Sudan, where a similar community ITN distribution programme was pro-rich, because poorer households were less likely to have heard about it [32]. It should be noted, however, that both settings are comprised of generally quite poor populations.

Community acceptability of the scheme was good, with close to 90% of beneficiaries reporting that the system was fair, the distances to obtain coupons and nets were short, the community agents were accessible, and the eligibility criteria were acceptable or good. Among the 94 households that requested a coupon but did not get one, only 15% said this was because the agent did not have any coupons; the remainder reported they either did not know why they did not get one, or because they did not meet the criteria, indicating that additional SBCC may be helpful in clarify the eligibility criteria to the beneficiary as well as to the community agents. The very low number of nets reported to have been obtained without a coupon (8) is also striking. The detailed tracking system used by ODDIT and CRS to document the flow of nets to individual religious leaders likely also contributed to a heightened sense of accountability. It suggests that the design of this community mechanism was able to draw strength from a social norm that this system should not be abused.

SBCC activities contributed significantly to the effectiveness of the programme. Families that received information about the scheme were more likely to have contacted a community agent to obtain a new net, and more likely have all family members sleeping under an ITN the night before. SBCC is an important component to any targeted distribution scheme and especially for a new program that requires the beneficiary to engage at multiple levels in the process. Programme planners should design SBCC activities that ensure community awareness of the scheme, provide clear information about eligibility criteria, and describe the steps of the process to request coupons and obtain ITNs, in addition to generalized messaging promoting ITN use.

The finding that ITN use was lower among children under 5 than among the general population is contrary to findings from other studies in Madagascar, where children under 5 are prioritized for ITN use [9, 33, 34]. In the 2016, 2013 and 2011 Malaria Indicator Surveys, under-5 ITN use was consistently 5–8% points higher than overall population ITN use [35–37], indicating that these results may be an anomaly.

Community-based distribution is challenging to implement, requiring training, ongoing supervision and strong logistics and supply chains not only for the ITNs but for the coupons and record-keeping supplies. Strong community networks are a prerequisite for community-based distribution; Madagascar has a history of community initiatives, and with the significant support provided through local NGOs [23, 38–42], but a pilot in a single district is quite different than regional or national scale-up. Costs of this pilot were not evaluated, but are crucial to consider, as previous research has hypothesized that at scale, community-based distribution may have higher costs per net delivered, due to smaller and more frequent deliveries to village level, and increased training, monitoring and supervision costs, compared to other channels [26]. In part due to the successful implementation of this pilot, Madagascar has included community distribution in its National Malaria Strategy, and is currently implementing community distribution in 6 districts [23]. In combination with free mass distribution campaigns, the community distribution is intended to replace damaged ITNs and to cover new sleeping spaces. It is essential to evaluate this programme at scale and over a longer period of implementation, to better understand how well the channel maintains ITN ownership and access in combination with mass campaigns, or indeed if no mass campaigns are implemented. Likewise, cost analyses and cost-effectiveness of larger-scale community distribution are needed to understand how this approach compares to implementation of repeated mass campaigns, and other combinations of distribution channels.

While this pilot was not designed to test whether community-based distribution could replace mass distribution, the evaluation of the pilot did show that community distribution can provide enough ITNs to sustain coverage 18 months after the last mass campaign. Indeed, the results indicate that the combination of a mass campaign with community distribution achieves sustained high coverage. The study also suggests that a community-based system has the potential to make ITNs available to the community without interruption. In principle, household demand for ITNs will reflect their needs, and a community-distribution scheme can supply ITNs when and where they are needed, potentially reducing oversupply if implemented as the primary ITN channel. However, without checks and balances, households may have opportunities to continuously request new free ITNs even if they are not needed, or may, in this system, be incentivized to not take care of their ITNs because new ones are continuously available. More research is needed to evaluate household ITN use and replacement behaviours in this type of on-demand system.

The largest limitation of this research is the short duration of the pilot implementation. A 9-month pilot is insufficient to fully test whether continuous systems can sustain ITN ownership and access over the long term; implementation periods of 3 or more years would provide more comprehensive answers to the research questions. A second limitation is that no baseline was conducted, due to inadequate funding and time constraints. The analysis excluding the community ITNs is an estimation; it must be acknowledged that households may have been more motivated to obtain additional ITNs through other channels, including markets or other commercial channels, had the community ITNs not been available. On the other hand, other studies have shown that 18 months after mass campaigns, ITN indicators fall as nets wear out and are discarded [28, 43–47]. We would not expect ITN ownership and population ITN access to remain above Roll Back Malaria target levels a year and a half after the mass campaign, as they did here.

## Conclusion

Household ITN ownership and population ITN access exceeded Roll Back Malaria target levels 18 months following a mass campaign and after a 9-month community distribution pilot in Toamasina II District in Madagascar. The pilot successfully provided coupons and ITNs to households requesting them, particularly for the least poor wealth quintiles, and the scheme was well perceived by communities. Further research is needed to determine whether community-based distribution can sustain ITN ownership and access over the long term, how continuous availability of ITNs affects household net replacement behaviour, and whether community-based distribution is cost-effective in combination with mass campaigns and/or other continuous channels.
